# Seasonal Variations in Fungal Communities on the Surfaces of Lan Na Sandstone Sculptures and Their Biodeterioration Capacities

**DOI:** 10.3390/jof9080833

**Published:** 2023-08-08

**Authors:** Paradha Nonthijun, Natasha Mills, Nantana Mills, Rujipas Yongsawas, Chakriya Sansupa, Nakarin Suwannarach, Churdsak Jaikang, Kannipa Motanated, Pattarasuda Chayapakdee, Surachai Jongjitngam, Nuttapol Noirungsee, Terd Disayathanoowat

**Affiliations:** 1 Department of Biology, Faculty of Science, Chiang Mai University, Chiang Mai 50200, Thailand; paradha_nonthi@cmu.ac.th (P.N.); natasha_mills@cmu.ac.th (N.M.); nantana_mills@cmu.ac.th (N.M.); rujipas_y@cmu.ac.th (R.Y.); chakriya_s@cmu.ac.th (C.S.); suwan.462@gmail.com (N.S.); pattarasuda.c@cmu.ac.th (P.C.); 2 Center of Excellence in Microbial Diversity and Sustainable Utilization, Chiang Mai University, Chiang Mai 50200, Thailand; 3 Toxicology Section, Department of Forensic Medicine, Faculty of Medicine, Chiang Mai University, Chiang Mai 50200, Thailand; churdsak.j@cmu.ac.th; 4 Department of Geological Sciences, Faculty of Science, Chiang Mai University, Chiang Mai 50200, Thailand; kannipa.motanated@cmu.ac.th; 5 Department of Thai Art, Faculty of Fine Arts, Chiang Mai University, Chiang Mai 50200, Thailand; surachai.j@cmu.ac.th

**Keywords:** seasonal variation, fungal communities, biodeterioration, sandstone

## Abstract

Environmental factors and climate are the primary factors influencing the microbial colonization and deterioration of cultural heritage in outdoor environments. Hence, it is imperative to investigate seasonal variations in microbial communities and the biodeterioration they cause. This study investigated the surfaces of sandstone sculptures at Wat Umong Suan Phutthatham, Chiang Mai, Thailand, during wet and dry seasons using culture-dependent and culture-independent approaches. The fungi isolated from the sandstone sculptures were assessed for biodeterioration attributes including drought tolerance, acid production, calcium crystal formation, and calcium precipitation. The results show that most of the fungal isolates exhibited significant potential for biodeterioration activities. Furthermore, a culture-independent approach was employed to investigate the fungal communities and assess their diversity, interrelationship, and predicted function. The fungal diversity and the communities varied seasonally. The functional prediction indicated that pathotroph–saprotroph fungi comprised the main fungal guild in the dry season, and pathotroph–saprotroph–symbiotroph fungi comprised the dominant guild in the wet season. Remarkably, a network analysis revealed numerous positive correlations among fungal taxa within each season, suggesting a potential synergy that promotes the biodeterioration of sandstone. These findings offer valuable insights into seasonal variations in fungal communities and their impacts on the biodeterioration of sandstone sculptures. This information can be utilized for monitoring, management, and maintenance strategies aimed at preserving this valuable cultural heritage.

## 1. Introduction

Chiang Mai, located in northern Thailand, has profound historical significance as the capital of the Lan Na Kingdom, epitomizing a long and illustrious heritage of cultural and traditional sculpture. The Lan Na sandstone sculptures, situated in a courtyard of Wat Umong Suan Phutthatham in Chiang Mai, were estimated to be built between 1400 and 1500 AD in the historical Lan Na areas that are now recognized as Phayao Province and moved to Wat Umong Suan Phutthatham in 1968 AD (Wimolyanmunee, personal communication, 10 February 2022). Most of these sculptures remain unexplored. It is plausible that the bases of some of these sculptures may contain inscriptions of ancient characters that hold archaeological and historical significance. Therefore, it would be unfortunate if these valuable pieces of cultural heritage deteriorated due to inadequate maintenance.

Sculptures suffer from deterioration as a result of weathering, which is caused by a combination of physical, chemical, and biological forces [1]. Microorganisms, including bacteria, fungi, algae, and lichen, act as the main factors causing severe damages in the conservation of cultural heritage due to their potential to cause biodeterioration [2]. For instance, fungi and their hyphae can penetrate stone and form non-uniform pits spanning up to centimeters in diameter, or cause damage via different pathways, such as the production of acids or digestive enzymes or direct physical damage [3,4]. These deterioration processes vary depending on the sculpting material used. Sandstone is among the materials commonly used for the construction of large temples and monuments. Notably, some of the most renowned cultural heritage sites, including Angkor Wat, Angkor Thom, and Prasat Preah Vihear, were constructed using primarily sandstone [1]. Sandstone is a sedimentary rock that is mainly composed of sand-sized particles such as minerals, lithic fragments, or organic materials. Terrigenous sandstone is primarily composed of quartz, feldspar, or lithic fragments, while accessory minerals make up a small percentage of the components. All components are lithified via compaction or bonded together via cement [5]. Sandstone is considered to be highly bioreceptive due to its rough and porous surface, which promotes microbial colonization, especially when combined with other environmental factors, such as the availability of water [2].

The occurrence and spread of biodeterioration on the surfaces of sculptures via the activities of microorganisms are believed to be associated with environmental factors such as pH, temperature, humidity, and nutrients across different seasons and locations [6,7]. In other words, the diversity and population of microbes are greatly influenced by changes in environmental factors. Seasonal variations in temperature and precipitation not only directly promote the degradation of building materials and the deterioration of monuments, but they also have significant influences on the structures and enzymatic activities of microbial communities [8,9]. Therefore, studying the seasonal variations in fungal communities on sandstone sculptures would help identify the dominant groups of fungi responsible for biodeterioration during different seasons.

This study investigated seasonal variations in fungal communities and their impacts on Lan Na sandstone sculptures. We compared fungal communities using samples collected from sandstone sculptures located at Wat Umong Suan Phutthatham during the wet and dry seasons of 2022 and 2023. We investigated differences in the diversity, structure, and function of the fungal communities by using culture-dependent and culture-independent approaches. This study illustrates the impacts of seasonal variations on microbial communities in cultural heritage environments. It reveals that microbes are exceptionally responsive to seasonal changes, and alterations in environmental conditions can significantly influence their community composition and metabolic activity. Consequently, these findings suggest the opportunity to customize protection strategies for different seasons to effectively mitigate risks of biodeterioration.

## 2. Materials and Methods

### 2.1. Sample Collection and Isolation

**Figure 1:**
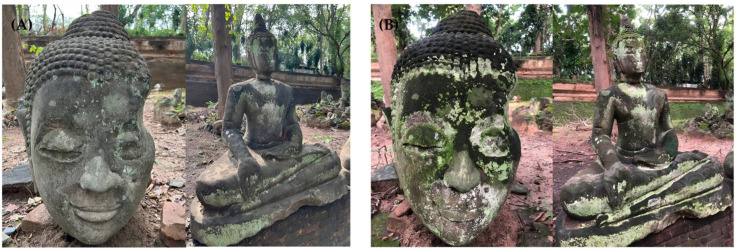
Visible biodeterioration on the surfaces of Lan Na sandstone sculptures at Wat Umong Suan Phutthatham, Chiang Mai, Thailand, in the dry season (**A**) and wet season (**B**). Photo taken by Paradha Nonthijun.

Microbial biofilm samples were collected from the surfaces of Lan Na sandstone sculptures exposed to an outdoor environment in a courtyard of Wat Umong Suan Phutthatham (18°46′35.66″ N 98°57′2.81″ E), Chiang Mai, Thailand, using the same sculptures in the wet season (August 2022) and dry season (February 2023) (Figure 1). The courtyard was surrounded by *Garuga pinnata*, *Xylia xylocarpa* (Dunal) Hook.f. & Thomson, *Anogeissus acuminata* (Roxb. ex DC.) Guili, and *Miliusa velutina* (Roxb.) W. Theob. Generally, sandstones are mainly composed of quartz and feldspar and contain small amounts of mica, calcite, clay minerals, and iron oxide [10]. Upon observing the Lan Na sandstone under a geological hand lens, it was identified as a medium- to coarse-grained massive sandstone. The grains range from sub-rounded to well-rounded and have a medium degree of sphericity. The majority of framework grains are quartz and feldspar. The sandstones were quarried around Doi Pha Kiang, which is located in the Tha Wang Thong and Mae Puem subdistricts of the Mueang district, Phayao province. These sandstones were from the Pong Klua Formation, which is equivalent to the Jurassic Phra Wihan Formation or Unit ms4 of the Khorat Group [11]. This formation is characterized by white, white-gray, yellowing, gray, gray-green, light brown, and reddish brown quartzitic and arkosic sandstones [12]. The nine replicates samples were scraped aseptically using a sterilized scalpel. Each collected sample was kept separately in a sterilized centrifuge tube. The DNA/RNA shield (Zymo Research) was added, and the samples were kept at −20 °C until DNA extraction. In August (the wet season), the relative humidity ranged between 85% and 90%, with a temperature of 25 °C. In February (the dry season), the relative humidity ranged between 65% and 70%, with a temperature of 17 °C (data obtained from https://www.tmd.go.th/forecast and https://th.weatherspark.com, accessed on 28 August 2022, 14 February 2023).

After collection, all samples were serially diluted tenfold with a 0.85% sodium chloride solution (NaCl) at a concentration ranging from 10^−1^ to 10^−5^. Then, 100 μL of each concentration was spread on dichloran rose bengal chloramphenicol (DRBC) agar to selectively encourage the growth of fungi. The plates were incubated at room temperature for 3–5 days. Then, the different characteristics of the hyphal tip from each plate were noted, and the tips were picked and placed on a new potato dextrose agar (PDA) plate until they emerged as a single colony.

### 2.2. Culture-Dependent Study

#### 2.2.1. Biodeterioration Study

##### Drought Tolerance

The isolates were cultured on a PDA containing sorbitol at concentrations of 0, 85, 175, 285, 405, 520, and 605 g/L. The plates were then incubated at room temperature for 3–5 days. After that, the growth of the fungi was observed.

##### Production of Organic Acids

The isolates were cultured in a potato dextrose broth (PDB) for 3–5 days, and the initial pH was measured. After 3–5 days of incubation, the final pH was measured. The isolates that had the lowest pH levels compared to the initial pH level were subjected to proton nuclear magnetic resonance (^1^H-NMR) spectroscopy at 500 MHz (Bruker NEO, Bruker Daltonics, Billerica, BA, USA) for organic acid determination. The Human Metabolome Database (HMDB) was used to identify the organic acids.

##### The Biomineralization of Calcium Carbonate

Calcium precipitation was screened using B4 agar plates of the following composition: 2.5 g of calcium acetate, 4 g of yeast extract, 10 g of glucose, and 15 g of agar per liter of deionized water. Then, 100 μL of fungal culture in PDB was spread on the B4 agar and incubated at room temperature for 21 days. After that, the incubated agar was cut into small pieces (measuring approximately 1 × 1 cm) and dried overnight at 60 °C. A scanning electron microscope (JEOL-JSM-IT300LV) was used to examine the calcium precipitation, and an energy-dispersive X-ray spectrometer (EDS) was used to analyze its chemical composition.

##### Calcium Carbonate Dissolution

The ability of the fungal isolates to solubilize calcium carbonate was determined by measuring their calcium solubilization. A medium of the following composition was used: 10 g of glucose, 5 g of calcium carbonate, and 15 g of agar per liter of deionized water. The plates were then incubated at room temperature for 3–5 days. After that, the clear zones (solubilization zones) were measured.

##### Siderophore Production

The isolates were cultured on a chrome azurol S-modified Guas No.1 (CAS-MGs-1) agar of the following composition: 900 mL of deionized water, 20 g of glucose, 1.0 g of potassium nitrite, 0.5 g each of sodium chloride, dipotassium hydrogen phosphate, and magnesium sulfate, and 15 g of agar. This was mixed with a CAS solution of the following composition: 60.5 mg of CAS, 72.9 mg of hexadecyltrimethylammonium bromide (HDTMA), 2.7 mg of iron (III) chloride hexahydrate, and 10 mM of hydrochloric acid. The sample was then incubated in the dark at room temperature for 21 days. After that, the change in the color of the media was observed.

#### 2.2.2. Molecular Identification of Fungal Isolates

The genomic DNA of the fungal isolates from selected colonies was extracted using a ZymoBIOMICS^TM^ DNA Miniprep Kit. For each DNA sample, amplification was conducted using a primer specific to the internal transcribed spacer region [13], namely ITS4/ITS5. In each PCR, the following components were used: 1.25 µL of each primer, 2.5 µL of 10X PCR buffer, 0.5 µL of 10 mM dNTP, 1.5 µL of 25 mM MgCl, 0.5 µL of Taq polymerase, 1.0 µL of DNA template, and distilled water adjusted to a total volume of 25 µL. The DNA from each sample was amplified according to the following cycling conditions: denaturation at 95 °C for 3 min, followed by 35 cycles of denaturation at 95 °C for 30 s, annealing at 55 °C for 40 s, extension at 72 °C for 30 s, and a final extension at 72 °C for 5 min. The reaction was then maintained at 4 °C. The PCR product was confirmed via gel electrophoresis. Sequencing was carried out using the dideoxy technique at Macrogen Inc., Seoul, Korea. MEGA11 was used to edit and align all of the sequences (version 11.0.10). These sequences were subjected to a BLAST search against the NCBI GenBank database. The organisms’ taxonomic classifications were assigned based on the first hit with the maximum sequence identity in the BLAST search.

### 2.3. Culture-Independent Study

#### 2.3.1. Preparation of Genomic DNA for Next-Generation Sequencing 

DNA was extracted using a ZymoBIOMICS^TM^ DNA Miniprep Kit. The concentration of the genomic DNA was assessed via a NanoDrop (a concentration of >10 ng/μL and purity of ~2.0 was required). Sequencing was performed using the Illumina MiSeq platform at Macrogen (Geumcheongu, Seoul, Republic of Korea).

#### 2.3.2. Next-Generation Sequencing (NGS) of DNA for Metagenomic Sequencing

The fungal communities in the samples were analyzed using Quantitative Insights Into Microbial Ecology 2 (QIIME2) software, version 2023.5. In QIIME2, all fungal DNA sequences were trimmed to remove the primer sequence at the internal transcribed spacer one (ITS1) region, using the forward primer ITS1F 3′-TCCGTAGGTGAACCTGCGG-5′ and the reverse primer ITS2R 3′-GCTGCGTTCTTCATCGATGC-5′ [14]. DADA2 was used to denoise the sequences, remove low-quality reads, and generate amplicon sequence variants (ASVs). The singletons were then removed, and the rarefaction curve was generated. The fungal taxa were assigned to the ASVs using a naive Bayes classifier trained on the UNITE database (version 8.2) at a confidence level of 95%. The proportions of fungi found in the sandstone sculptures were shown in a stacked bar format.

#### 2.3.3. Alpha Diversity and Beta Diversity Analysis

The alpha diversity, including the Simpson index, Shannon index, and Chao1 index, was analyzed using PAST software, version 4.11. The results were then visualized as boxplots. For a beta diversity analysis, a one-way PERMANOVA was conducted using the Bray–Curtis similarity index to assess significant differences in fungal diversity between the wet and dry seasons among the Lan Na sandstone sculptures. Additionally, a non-metric multidimensional scaling (NMDS) plot was generated using PAST software, version 4.11 [15].

#### 2.3.4. Network Analysis

An analysis of the networks among the fungal communities was carried out using R studio software with the packages Hmisc and Vegan to generate the co-occurrence and co-exclusion networks of the OTUs. Gephi 0.92 was used to visualize the correlations, which were generated for a Fruchterman–Reingold plot [16].

#### 2.3.5. Functional Prediction Analysis

FUNGuild software was used to predict the metagenome functional genotypes of the microbiota colonizing the Lan Na sandstone sculptures [17].

#### 2.3.6. DNA Sequence Deposition

All fungi sequences were deposited in the GenBank database (accession numbers OQ283805—OQ283818 for fungi isolates collected in the wet season and OR048738—OR048741 for fungi isolates collected in the dry season). The raw sequences are available for bioinformatics study in the National Center for Biotechnology Information (NCBI) under the BioProject accession number PRJNA977821.

## 3. Results

### 3.1. Fungal Isolates and Drought Tolerance

In total, eleven pure fungal cultures were isolated from the sandstone sculptures (seven isolates from sandstone sculptures in the wet season, WG, WI, WJ, WK, WL, WM, and WO, and four isolates from the sandstone sculptures in the dry season, DF1, DF3, DF5, and DF8). Using PDA, all fungal isolates were examined for biodeterioration abilities. The agar was mixed with sorbitol in eight concentrations to test the drought tolerance of the fungal isolates collected from sandstone sculptures in the dry season only. All the isolates demonstrated an ability to tolerate drought, especially isolate DF1, which exhibited tolerance to the highest concentration of sorbitol (Table S1).

### 3.2. The Production of Organic Acids

**Figure 2:**
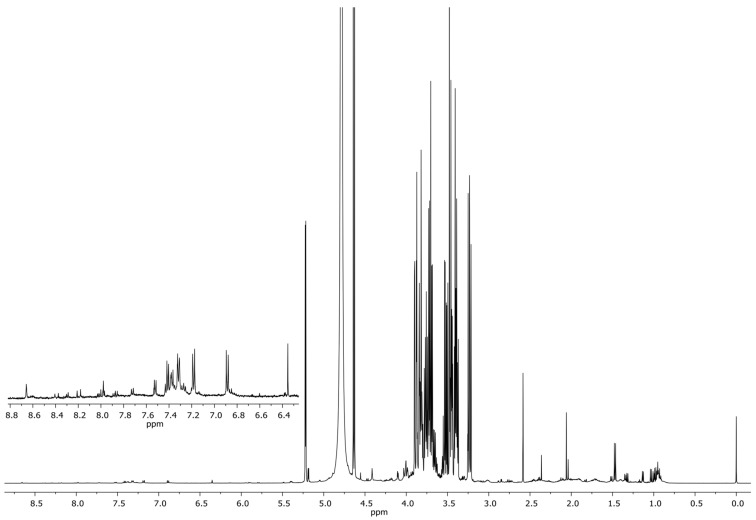
The ^1^H-NMR spectra of the broth medium (PDB) containing *Pestalotiopsis* sp. after 5 days of incubation, when TSP = 0.00, acetic acid = 1.91, malic acid = 2.36, succinic acid = 2.40, citric acid = 2.53, lactic acid = 4.10, fumaric acid = 4.79, and oxalic acid = 6.39 ppm, respectively.

Following their incubation in a broth medium (PDB), the pH levels of the isolates were considered (Table S2). After that, the isolates with the lowest final pH levels were subjected to NMR spectroscopy. According to the NMR data, the fungal isolates that demonstrated biodeterioration abilities were capable of producing organic acids, including malic acid, acetic acid, citric acid, lactic acid, fumaric acid, succinic acid, and oxalic acids. This result found that the representative isolates were able to produce all the referenced organic acids (Figure 2).

### 3.3. The Biomineralization and Dissolution of Calcium Carbonate

**Figure 3:**
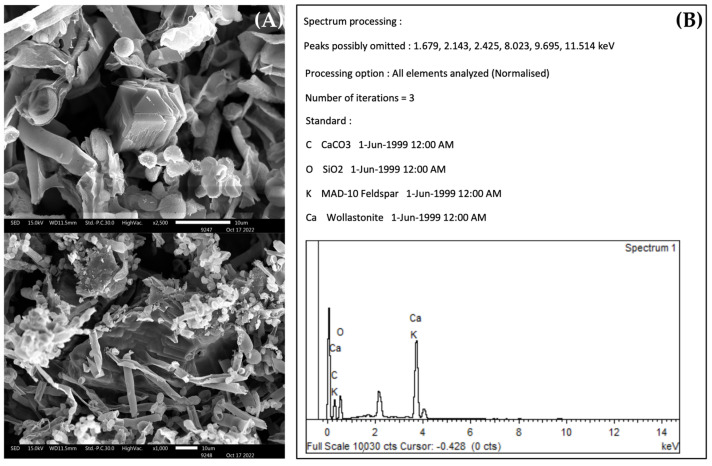
An example of calcium crystal formation by *Aspergillus* sp. (**A**) and an EDS analysis of *A. niger* (**B**). In (**B**), C = calcium carbonate, O = silicon dioxide, K = MAD-10 feldspar, and Ca = wollastonite.

The abilities of the fungal isolates from the two seasons to precipitate calcium and form crystals were examined using a scanning electron microscope (SEM). In the wet season, three fungal isolates, WF9, WF13, and WF15, could form calcium crystals on B4 agar after being incubated for 21 days, as shown in Figure 3. In the dry season, all isolates had the ability to form calcium crystals. In contrast, the ability to dissolve calcium carbonate was rarely observed. Only two fungal isolates from the wet season, WI and WL, and isolate DF1 from the dry season produced clear zones around their colonies (Figure 3). The resultant clear zones had diameters of 14 mm for WI and 14.7 mm for WL.

### 3.4. Siderophore Production

**Figure 4:**
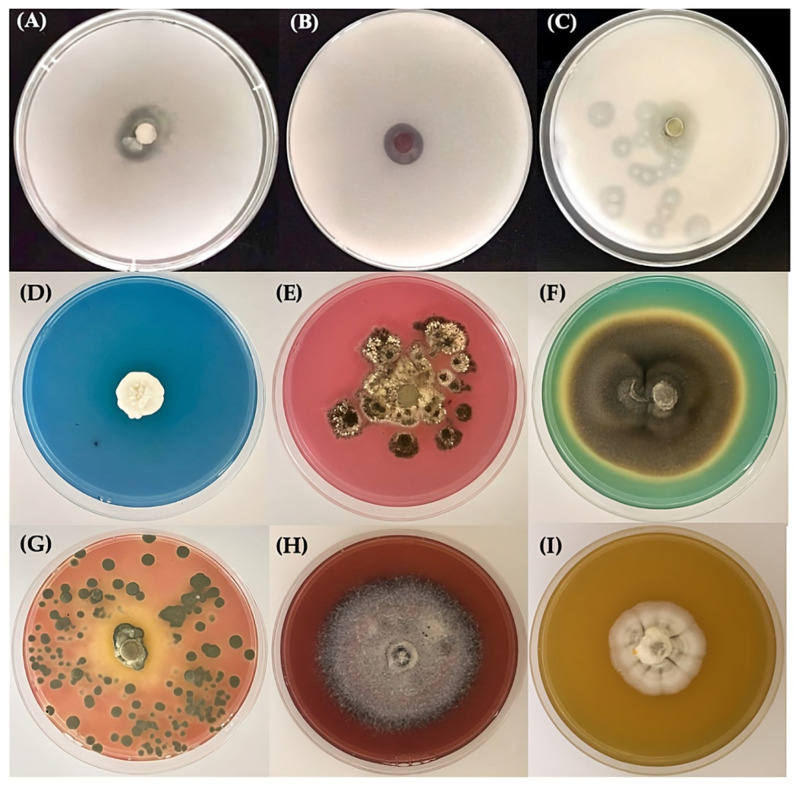
Clear zones on plates of calcium carbonate agar containing fungal isolates from the wet season (**A**,**B**) and dry season (**C**) and examples of different changes in color of chrome azurol S-modified Guas No.1 (CAS-MGs-1) agar after the incubation of the fungal isolates (**D**–**I**).

The fungal isolates’ ability to produce siderophores was examined by growing the fungi on chrome azurol S-modified Guas No.1 (CAS-MGs-1) agar and incubating them in the dark at room temperature for 21 days. All fungal isolates except DF8 demonstrated positive reactions, changing the color of the agar from blue to pink, orange, red, green, and yellow (Figure 4). The different changes in color of the CAS-MGs-1 agar arose from the production of different types of siderophores. In addition, the intensities of the colors may be related to the concentrations of the siderophores [18].

### 3.5. The Molecular Identification of Fungal Isolates

All fungal isolates were identified using the ITS1-5.8S-ITS2 region. The genomic DNA of the fungi isolates was extracted and sequenced. The isolates belonged to six different genera: *Fusarium*, *Penicillium*, *Aspergillus*, *Trichoderma*, *Phoma*, and *Pestalotiopsis* (Table S3).

### 3.6. Fungal Community Structures in Sandstone Sculptures in Wet and Dry Seasons, Characterized via a Culture-Independent Molecular Technique

**Figure 5:**
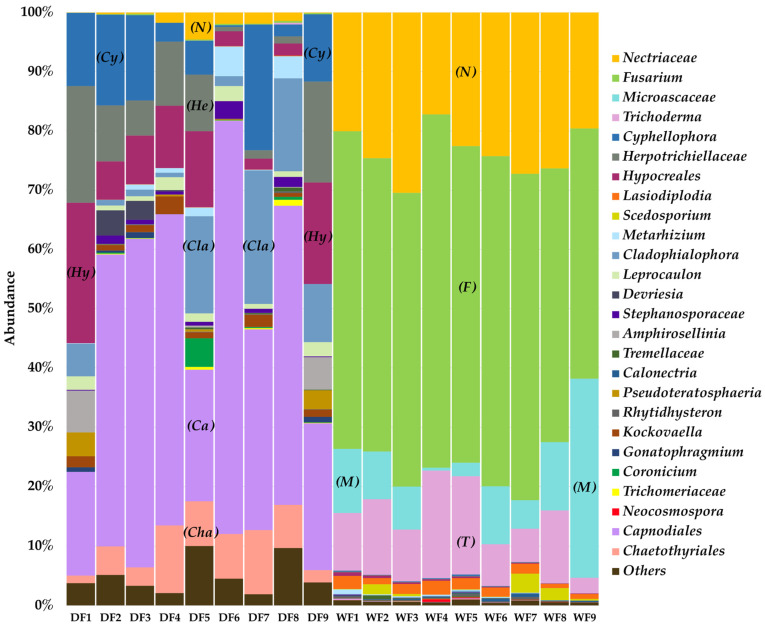
Fungal proportions in microbial biofilm from the dry season (DF) and wet season (WF); the name of the bar section in each color represents the abundance of the fungal group in each season. *N = Nectriaceae*, *F = Fusarium*, *M = Microascaceae*, *T = Trichoderma*, *Cy = Cyphellophora*, *He = Herpotrichiellaceae*, *Hy = Hypocreales*, *Cla = Cladophialophora*, *Ca = Capnodiales*, and *Cha = Chaetothyriales*.

The fungal communities were analyzed based on a total of 255 genera, and the data were edited to 26 genera. The most abundant fungal group among the samples from the dry season was Capnodiales, representing 43.58% of the community, followed by *Cyphellophora*, which contributed to 9.74%. In contrast, *Fusarium* was the dominant genus among the samples from the wet season, accounting for 51.65% of the community. Additionally, Nectriaceae and Microascaceae contributed 23.56% and 9.85%, respectively, (Figure 5). An analysis at the species level showed that the members of *Fusarium* present in the wet season were *F.solani* and *F. albosuccineum*.

### 3.7. Alpha and Beta Diversity

**Figure 6:**
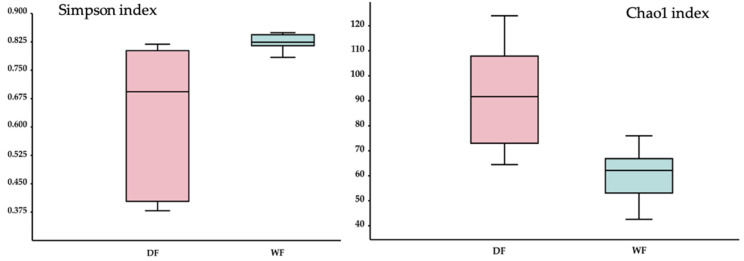
Boxplots of the alpha diversity of the fungi according to the Simpson and Chao1 indexes in samples collected from the dry season (DF) and wet season (WF). The boxes represent the interquartile range (IQR) between the first and third quartiles (25th and 75th percentiles, respectively), and the horizontal line inside the box defines the median.

For the fungal communities, the Simpson diversity index communities showed a significant difference (*p* < 0.05) between the dry season and wet seasons, with a higher level of diversity observed in the dry season. Similarly, the Chao1 index also exhibited a significant difference (*p* < 0.05) between the dry and wet seasons, with higher numbers of observed species in the dry season (Figure 6).

**Figure 7:**
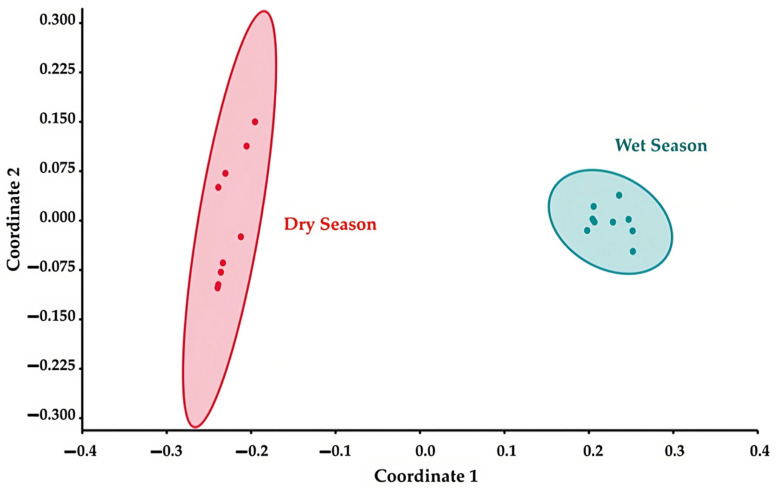
The NMDS plot shows the differences in fungal taxa at the ASV level and quantity among the samples from the two seasons. The red dots represent samples from the dry season, while the green dots represent samples from the wet season.

The beta diversity was assessed using the Bray–Curtis index to examine the differences between the communities of microorganisms in the two seasons. The result reveals significant distinctions between the fungal communities in the two seasons (PERMANOVA, *p* = 0.0001). These differences in the fungal communities between the seasons were visualized in an NMDS plot based on the Bray–Curtis index (Figure 7).

### 3.8. Network Analysis

**Figure 8:**
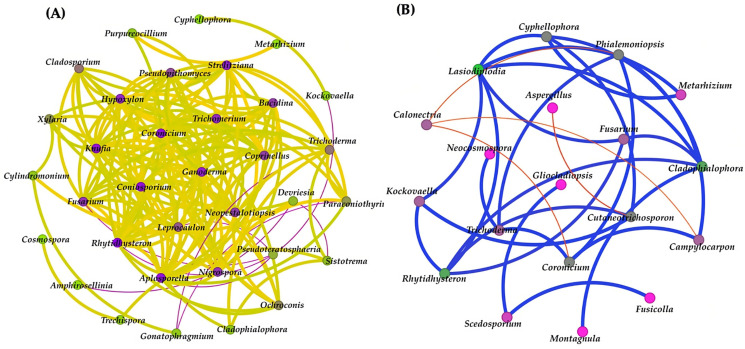
(**A**) The network analysis among the samples in the dry season, showing positive (yellow line) and negative (purple line) correlations; (**B**) the network analysis among the samples in the wet season, showing positive (blue line) and negative (red line) correlations (**B**) between fungal taxa in the biofilms on the surfaces of the Lan Na sandstone sculptures.

In a comparison of the correlation network analysis results between the two seasons, the result for the dry season found only one community that showed a positive correlation (represented by a yellow line), and negative correlations (represented by purple lines) were present for 34 fungal identities and 165 reactions among them. The result suggests that many fungal taxa have positive correlations with each other, while *Pseudoteratosphaeria* were the most negatively correlated with other fungi (Figure 8). In addition, the result from the wet season also found only one community that showed a positive correlation (represented by blue line), and negative correlations (represented by red lines) were present for 19 fungal identities and 18 interactions. According to the result from the wet season, *Cladophialophora* and *Trichoderma* were the most positively correlated with other fungi in the sample in contrast to *Calonectria*, which were negatively correlated with other fungi (Figure 8).

### 3.9. Functional Prediction Analysis

**Figure 9:**
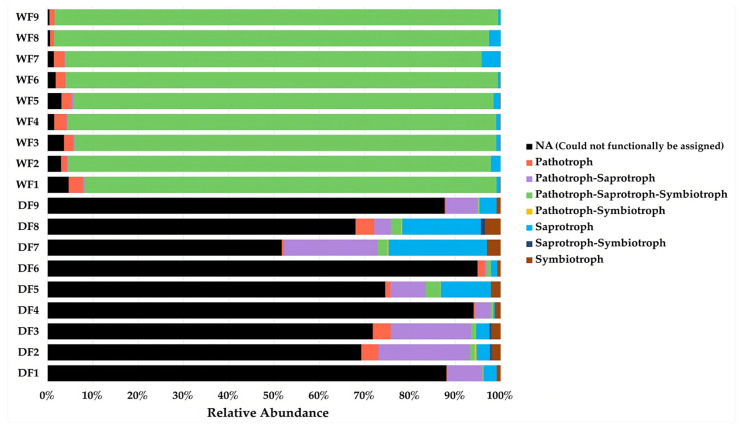
Guild variations in the fungal communities from the dry season (DF) and wet season (WF).

The functional variation in the fungal guilds, which were defined based on trophic modes, were evaluated among the samples between the two seasons. In the dry season, most of the fungal ASVs could not functionally be assigned to guilds (NA), with a relative abundance of 77.77%. Pathotroph–saprotroph fungi represented the second largest guild (9.88%), followed by saprotroph fungi (7.91%), while pathotroph–saprotroph–symbiotroph fungi comprised the largest guild found in the communities from the wet season, with a relative abundance of 93.95% (Figure 9).

## 4. Discussion

Ancient sandstone buildings hold immense value and significance in human society. They are not only treasured architectural structures, but also serve as integral components of a community’s identity, history, and cultural expression. Regrettably, the majority of these ancient sandstone buildings underwent severe deterioration, leading to irreversible loss and damage. The deterioration of ancient sandstone buildings can occur due to a combination of physical and biological factors. This study provides insight into the structures and biodeterioration activities of fungal communities associated with the Lan Na sandstone sculptures at Wat Umong Suan Phutthatham, Chiang Mai, Thailand, during two different seasons (the wet season and the dry season). The dry season is characterized by an average temperature of 17 °C and a relative humidity of 65–70%. The wet season is characterized by an average temperature of 25 °C and a relative humidity of 85–90%. These differences may influence the structures of the microbial communities and their activities.

The results of the biodeterioration assay show that the fungal isolates from the samples acquired in the dry season had the ability to tolerate dry conditions or conditions with low levels of water activity. This ability can have a significant impact on the biodeterioration of sandstone sculptures, as it is indicated that fungi capable of thriving in conditions with low water activity levels may directly affect the sculptures or create a suitable environment for the growth of other microorganisms that can cause damage [4,19].

Most of the fungal isolates could produce organic acids and secrete them into the surrounding environment [20]. Fungi are known to produce acidic metabolites in both a medium containing glucose and in various building materials [21]. These organic acids include malic acid, acetic acid, citric acid, lactic acid, fumaric acid, and succinic acid [22,23,24]. These acids can act as chelating agents, which have the ability to dissolve the positive ions of minerals such as Ca, Si, K, and Fe, which are present in the framework grains, matrix, or the cement of sandstone [5]. Organic acids are involved in both calcium precipitation and the calcium solubilization processes [20]. Fungi have the ability to release positive ions from minerals and subsequently precipitate them as secondary minerals, such as calcium oxalate (CaC_2_O_4_) or calcium carbonate (CaCO_3_), onto stone surfaces [24]. Oxalate production is involved in the biodeterioration of rock and mineral substrates, as well as the alteration and decay of cultural heritage [25]. From our results, *Aspergillus niger* and *Fusarium oxysporum* have the abilities to dissolve calcium carbonate and precipitate calcium in the form of wollastonite. This finding is consistent with the study by Yu et al., 2021 [26], which demonstrated that *A. niger* facilitated the weathering of wollastonite and was also capable of crystallizing wollastonite. The dissolution and precipitation of calcium associated with fungal growth can lead to the formation of calcium crystals. These calcium crystals have the potential to cause damage to sandstone by exerting increased pressure beneath the surface layers, ultimately resulting in cracking [20,22].

Most of the fungal isolates were able to produce siderophores. Changes in color and the depth of color may be related to the type and concentration of siderophores [18]. Siderophores are low-molecular-weight (500–1000 Da) iron-chelating ligands that are synthesized by microorganisms such as bacteria and fungi [27]. Generally, siderophores are classified into two structural groups, hydroxamates and catecholate compounds, which have high specificities for chelating or binding iron. Siderophores are usually produced and secreted in environments with low concentrations of iron. Iron is an essential element for nearly all organisms; it is used in various processes, including enzyme synthesis, electron transport, and DNA synthesis [28]. A series of papers reported the production of siderophores by several fungi, which relate to our results. In the first report [29], it was found that fungi belonging to the genera *Penicillium* and *Aspergillus* have the ability to produce hydroxamate-type siderophores, which can induce a color change from blue to orange in CAS-MGs-1 agar. A second report found that most *Aspergillus* fungi, such as *A. nomius*, can produce hydroxamate-type siderophores, which cause an orange color in agar [30]. In addition, *A. niger* and *Trichoderma asperellum* turned the color of agar from blue to pink due to their ability to produce catecholate-type siderophores, similar to the results in [31]. For *Fusarium solani*, *F. eqiseti*, and *F. oxysporum*, it was reported in [32,33,34] that fungi belonging to the genera *Fusarium* can produce hydroxamate-type siderophores in three forms, including ferricrocin, ferrichrome C, and malonichrome, which turned the blue color of agar into a clear orange or yellow. Unfortunately, there is no report on the production of *Phoma destructiva* by siderophores and the change in color of CAS-MGs-1 agar from blue to green. However, it was reported that *Phoma destructiva* can produce petasol and phomenone, which are phytotoxic secondary metabolites from fungi [2,35]. Perhaps these phytotoxins may have structures similar to those of siderophores and will react with the iron in the complex of the dye chrome azurol S to change the color of the medium to green. In addition, it was reported that *Phoma* spp. are able to penetrate rock material via hyphal growth and biocorrosive activity due to their excretion of organic acids or via the oxidation of mineral-forming cations, preferably iron and manganese [36]. The ability to produce siderophores and use them as chelators is interesting because sandstones are mainly composed of quartz or feldspars, whereas accessory minerals such as iron are minor components that fill in the spaces between these framework grains as a matrix [5,37]. Therefore, the fungal production of siderophores may lead to the biodeterioration of sandstone.

Capnodiales was the dominant group among the samples from the dry season. These are plant and human pathogens, endophytes, saprobes, and epiphytes. Some of them are lichenized and occur on stone or as a parasite on fungi [3]. Capnodiales is considered to be part of the group of microcolonial black fungi (MCBF) and rock-inhabiting fungi (RIF), which have the ability to grow in extreme environments such as hot and cold deserts, saltpans, acidic and hydrocarbon-contaminated sites, and on the surfaces of rocks [38,39]. In addition, they are associated with a variety of human skin diseases [40]. Therefore, these microorganisms are likely contaminants from visitors. They damaged the stones via hyphal penetration [41] and the production of extracellular polysaccharides [42]. The visible deterioration of sandstone associated with fungi of the family Capnodiales consists of pitting, a green patina, and discoloration [43]. *Cyphellophora* is a genus of black yeast-like fungi [44]; some species of them are included in the rock-inhabitant black fungi group and are known for their ability to grow in extreme environments. For instance, *Cyphellophora olivacea*, which are pigment producers, have pigmented aerial mycelium and/or reproductive structures that cause the deterioration of rocks [45]. In the wet season, *Fusarium* was the most abundant genera in the samples. *Fusarium* belong to the phylum Ascomycota, the largest fungal phylum. They are the most successful colonizers of soil and rocks because they are highly adaptable [46]. It was reported that *Fusarium* were found on architectures and sculptures built from various types of stone or other materials [47,48]. Several studies reported the presence of *Fusarium* on many pieces of cultural heritage around the world; the authors of [49] investigated 15 deteriorating sites at Gwalior Fort in India and found *Fusarium* on the historic monument site. According to the report in [50], *F. solani* was found on a sandstone castle at Angkor Wat in Cambodia and in the Painted Cave of Lascaux in France [51], and *F. oxysporum* was found on Dharmarajika, Taxila, Pakistan [52], on the historic buildings of Havana, Cuba [53], and on Mohamed Ali Palace, Cairo, Egypt [54]. *F. oxysporum*, *F. solani*, and *F. albosucciniem* are representatives of soilborne pathogens and were reported in a group of plant pathogens [55,56,57]; they can be found in soil and plants as well. Regarding the sample site of this study, the sandstone sculptures are located in open areas and are in close contact with plants and soil. Therefore, it is possible to find *Fusarium* in large proportions.

In this study, we used culture-dependent and culture-independent approaches to analyze the fungal communities and their associations with the biodeterioration of the Lan Na sandstone sculptures during two different seasons. An investigation of the fungal communities via Illumina sequencing (MiSeq) showed that the fungal communities were significantly different between the two seasons. The results of the alpha diversity index assessment indicate that the samples from the dry season scored higher in all indexes than the samples from the wet season. The dry season is associated with a decrease in moisture content, which can limit the availability of microorganisms. Some fungal groups, being able to adapt to drier conditions, may have a competitive advantage during this period. As a result, the reduced competition for resources during the dry season can allow certain fungal species to thrive and contribute to an increase in fungal diversity. Furthermore, the beta diversity analysis pointed out differences in fungal communities among the samples from the two seasons. In the dry season, the dominant type of fungi that we found was a group of rock-inhabiting fungi (RIF), which was more present than in the wet season. RIFs are considered oligotrophs that can survive on rock surfaces. These fungi perform excellently in a wide range of temperature extremes and under conditions of irradiation, osmotic stress, and desiccation [58,59]. Although the dry season in Thailand is not very dry when compared to Western countries, it is enough to cause a fluctuation in the temperature between the two seasons. The average temperature during the day at the sample site is around 17 °C, which agrees with the 2022 study by Liu et al. [58]. RIFs have a minimum temperature for growth below 20 °C. In the wet season, the proportion of fungi in the plant pathogen group was clearly higher than in the dry season due to the ambient temperature and humidity. The growth of many pathogens is favored by high humidity and temperature, with optimum ranges between 20 and 30 °C [60]. The average temperature at the sample site is around 25 °C, so it is suitable for the growth of plant pathogens. There is a possibility that rain possibly changed the fungal communities on the sandstone sculptures. While the rain is falling, water seeps into different parts of the plant, causing the plant pathogens that colonized the plant to be washed down and re-colonized on the surfaces of the sculptures. Therefore, plant pathogens were found in a large proportion.

The network analysis of the dry season samples showed that many fungal taxa have positive correlations with each other. For example, *Cladosporium* an*d Knufia*, which can produce extracellular melanin–polysaccharide complexes, which are compounds belonging to the group of extracellular polymeric substances (EPSs) [61,62]. EPSs lend stability to the biofilm by mediating cellular aggregation and bringing the cells in close contact with the substrate via adhesion [63], encouraging themselves and fungi to attach more strongly to the substrate and deteriorating it at a fast rate. In addition, it was reported that *Xylaria* were predominantly identified from mycelium particles of a biofilm biomass on the surface of a Mayan building [64]. The result indicates that the fungal taxa have positive relationships with each other in cases of the production of secondary metabolites and penetration by hyphae. In terms of *Pseudoteratosphaeria*, information about its functions or relations with other fungi is still scarce, but information indicated that *Pseudoteratosphaeria* are closely related to *Teratosphaeria* [65], which are primarily known as plant pathogens [66] and are not typically associated with causing direct damage to other fungi. However, in complex ecological interactions, various fungi can interact with one another, sometimes in competitive or antagonistic ways. It is possible that *Teratosphaeria* species could indirectly affect other fungi by competing with them for resources or by producing metabolites that inhibit their growth. In the wet season, *Cladophialophora* and *Trichoderma* were the most positively correlated with other fungi in the sample. Both were reported as rock-inhabiting fungi (RIF) [67,68]. *Cladophialophora* is a genus of black yeast-like fungi, which have the ability to produce melanin pigment to protect themselves and other fungi against environmental factors [69]. The production of melanin is determined by the availability of nutrients and minerals, UV radiation, temperature, and other environmental factors; it provides protection from excessive environmental radiation (UV radiation and X- and γ-rays) and chemical stressors [70]. Moreover, melanin can cause the absorption of solar radiation, thereby increasing the temperature of the surface of sandstone and causing a wet–dry cycle [71], which induces cracking and reduces the strength of the sandstone. On the other hand, *Calonectria* were the most negatively correlated with other fungi in the sample. There were reports that the *Calonectria* genome possesses a surprising number of secondary metabolism backbone enzyme genes involved in toxin biosynthesis [72], which may have inhibitory effects on other fungi. One example is *Calonectria ilicicola*, which produces a compound called ilicicolin that was reported as a virulence factor [73]. This could explain why *Calonectria* are negatively correlated with other fungi. The results of the network analysis show that seasonal variation has an impact on fungal networks, and the fungal correlation in the dry season is more complex than in the wet season.

FUNGuild is a software tool used for ecological analysis and the interpretation of fungal community data. It provides insights into the functional roles of fungal taxa within an ecosystem. Fungi have various trophic modes and functional guilds, and different trophic modes and functional guilds have different functions [74]. According to the result of this study, saprotroph fungi, including Microascacene, Herpotrichiellaceae, Stephanosporaceae, Tremellaceae, Tricomeraceae, Capnodiales, *Cyphellophora*, *Lasiodiplodia*, *Scedosporium*, *Cladophialophora*, *Leprocaulum*, *Devrisla*, *Amphirosellinia*, *Calonectria*, *Pseudoteratosphaeria*, *Rhytidrysteron*, *Kockovaella*, *Gonatophragmium*, *Coronicium*, and *Neocosmopora*, and pathotroph–saprotroph fungi, including Nectriacenae, Hypocreales, *Fusarium*, and *Trichoderma*, were found in all the fungal communities in both the dry and wet seasons, especially the dry season, while *Metarhizium*, which belongs to the pathotroph–saprotroph–symbiotroph group, was found in a large proportion in the wet season. These results indicate three main groups of fungi in the communities: saprotroph, pathotroph–saprotroph, and pathotroph–saprotroph–symbiotroph fungi. Saprotrophic fungi are a group of fungi that obtain nutrients by decomposing dead organic matter through the production of enzymes such as cellulases, β-glucosidases lignin peroxidases, and laccases [75]. Laccase enzymes are well known for the decomposition of lignocellulosic plant biomass materials in nature. Interestingly, there were reports that laccases are involved in the deterioration of black slates via bio-weathering processes [76]. Slate has a high content of organic carbon in form of kerogen, and its structure is similar to lignin; therefore, it is susceptible to laccase enzymes [76,77]. Hence, this can be an indicator that the ability of saprotrophic fungi to produce enzymes may be one of the causes of biodeterioration. Pathotroph–saprotroph fungi comprise a pathogenic group, i.e., they can cause diseases or infections in other organisms, including plants, animals, and humans. In cases of deteriorated stone, the most frequently reported pathogens that are involved in the biodeterioration of cultural heritage objects are plant pathogens [78,79] due to their activities. When they colonize rocks, they can induce deterioration by secreting organic acids and siderophores or by producing melanin compounds [80]. In the wet season, the group of fungi was changed, and the relative abundance of pathotroph–saprotroph–symbiotroph fungi increased obviously, while the relative abundance of pathotroph–saprotroph fungi was decreased. Symbiotrophic fungi, including mycorrhiza fungi, are a group of fungi that form mutually beneficial relationships with plants [81] and lichens, which are commonly recognized as symbiotic associations of fungi and chlorophyll-containing partners, either green algae or cyanobacteria [82]. Mycorrhiza fungi were often found in the wet season because the climate conditions in the wet season are suitable for plant growth. Relating to the results reported in [83], the relative abundances of brown rot fungi and plant pathogens decreased in the wet season, and the relative abundances of mycorrhizal fungi increased. Therefore, our results suggest that seasonal variations had significant effects on fungal community structures.

The results achieved using conventional methods and metagenomics enable us to identify the dominant groups of fungi and their biodeterioration activities. The fungi in the phylum Ascomycota, including *Aspergillus*, *Fusarium*, *Penicillium*, and *Trichoderma*, were the dominant groups in the sample. Interestingly, the actual damage observed on the sandstone sculptures in Wat Umong Suan Phutthatham was similar to the damage caused from the biodeterioration processes of these fungi, such as the emergence of bio-patinas, cracking, and discoloration [84,85]. Therefore, the production of organic acids, the secretion of siderophores or secondary metabolites, and the formation of calcium oxalate by these fungi are potential causes of the biodeterioration observed on the Lan Na sandstone sculptures. Importantly, seasonal variations had significant effects on the fungal communities. This study requires further in-depth research and information to achieve a deeper understanding of the fungal biodeterioration capacities of the biodeterioration processes. 

## 5. Conclusions

This study demonstrated the effects of seasonal variations on the fungal communities and the effects of their biodeterioration capacities on sandstone sculptures. The fungal diversity and communities in the sample from the sandstone sculptures differed between two seasons. These observed differences strongly indicate that seasonal variations have significant impacts on fungal communities and their biodeterioration activities on stone surfaces. Due to the differences in environmental conditions in ecosystems, the fungal diversity in the communities of fungi in the dry season was higher than that in the wet season, and the functional group was more diverse in the dry season than that in the wet season as well. Moreover, we found the different network correlations among the fungi in each season that allowed the sandstone to deteriorate. In order to effectively preserve this valuable site of cultural heritage, it is crucial to conduct thorough and in-depth investigations that can provide insights for long-term maintenance strategies.

## Data Availability

Publicly available datasets were analyzed in this study. These data can be found under the BioProject accession number PRJNA977821 and the accession numbers OQ283805—OQ283818 for fungi isolates in the wet season and OR048738–OR048741 for fungi isolates in the dry season.

## Supplementary Materials

The following supporting information can be downloaded at: https://www.mdpi.com/article/10.3390/jof9080833/s1, Table S1: Growth of fungal isolates on PDA agar mixed with sorbitol in each concentration; Table S2: pH values of fungal isolates after 5 days of incubation; Table S3: Identification of isolated fungal strains based on 18S rRNA gene sequence analysis and their close relatives published in NCBI databases.
