# Supplementary material for: Seasonal Variations in Fungal Communities on the Surfaces of Lan Na Sandstone Sculptures and Their Biodeterioration Capacities
Source: J Fungi (Basel). 2023 Aug 8;9(8):833. doi: 10.3390/jof9080833 (PMC10455195; doi:10.3390/jof9080833)
Supplement: Supplementary file 1 [file jof-09-00833-s001.zip › jof-2475525-supplementary.pdf]

Table S1. Growth of fungal isolates on PDA agar mixed with sorbitol in each concentration.

Isolates	Concentration of Sorbitol (g/L)						
	0	85	175	285	405	520	605
DF1	+	+	+	+	+	+	+
DF3	+	+	+	+	+	-	-
DF5	+	+	+	+	+	-	-
DF8	+	+	+	+	+	-	-

Note: + means fungi can growth on PDA agar; - means fungi cannot growth on PDA agar.

Table S2. pH value of fungal isolates after incubation for 5 days.

Isolates	Initial pH	Final pH
WF7	4.49	4.12 ± 0.122
WF8	4.49	3.91 ± 0.012
WF9	4.49	4.03 ± 0.017
WF10	4.49	3.67 ± 0.026
WF11	4.49	3.72 ± 0.067
WF12	4.49	3.50 ± 0.015
WF13	4.49	3.77 ± 0.036
WF15	4.49	4.02 ± 0.032
DF1	4.47	4.13 ± 0.015
DF3	4.47	3.98 ± 0.025
DF5	4.47	4.00 ± 0.035
DF8	4.47	3.96 ± 0.062

Note: WF ; isolates from sandstone sculpture in wet season. DF ; isolates from sandstone sculpture in dry season.

Table S3. Identification of isolated fungal strains based on 18S rRNA gene sequence analysis and their close relative published in NCBI databases.

Isolate	Description	Query Cover-age	Percent Identity	Accession Number
WG	*Penicillium citrinum* isolate NJC70 internal transcribed spacer 1	100 %	100 %	OQ283805<br>OQ283806
WI	*Aspergillus niger* isolate RMUAN40 internal transcribed spacer 1	100 %	100 %	OQ283807<br>OQ283808
WJ	*Fusarium solani* isolate sample47 internal transcribed spacer 1	100 %	99.78 %	OQ283809<br>OQ283810
WK	*Fusarium equiseti* isolate C1-47 small subunit ribosomal RNA gene	100 %	100 %	OQ283811<br>OQ283812
WL	*Fusarium oxysporum* isolate NJ3256 internal transcribed spacer 1	100 %	100 %	OQ283813<br>OQ283814
WM	*Aspergillus nomius* strain 110P internal transcribed spacer 1	100 %	100 %	OQ283815<br>OQ283816
WO	*Trichoderma asperellum* isolate Tr17 small subunit ribosomal RNA gene	100 %	100 %	OQ283817<br>OQ283818
DF1	*Penicillium* sp. Y28 18S ribosomal RNA gene, partial sequence	100%	100%	OR048738
DF3	*Phoma destructiva* gene for 18S rRNA, partial sequence, isolate: MUCC0064	99%	100%	OR048739

DF5	*Aspergillus niger* small subunit ribosomal RNA gene, partial sequence	99%	99%	OR048740
DF8	*Pestalotiopsis maculans* for 18S rRNA, partial sequence, strain: CBS 322.76	99%	100%	OR048741
